# Supplementary material for: Inhibition of Th2 Differentiation Accelerates Chronic Wound Healing by Facilitating Lymphangiogenesis
Source: Biomedicines. 2025 Apr 24;13(5):1026. doi: 10.3390/biomedicines13051026 (PMC12109103; doi:10.3390/biomedicines13051026)
Supplement: Supplementary file 1 [file biomedicines-13-01026-s001.zip › biomedicines-3549667-supplementary.pdf]

Inhibition of Th2 Differentiation Accelerates Chronic Wound Healing by Facilitating Lymphangiogenesis

Bracha L Pollack[1], Jeremy Torresi[1], Geoffrey Hespe[1], Gopika Ashokan[1], Jinyeon Shin[1], Babak J Mehrara[1] and Raghu P Kataru[1]*

Supplementary Data

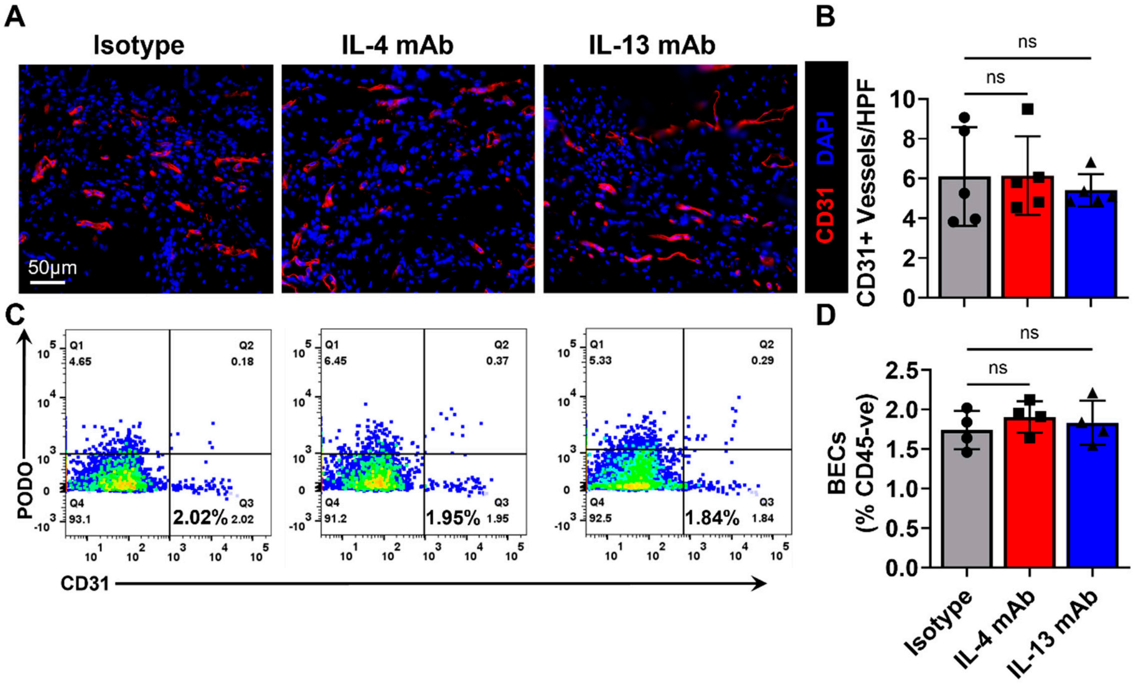

Supplementary Figure S1. Inhibition of Th2 differentiation causes no changes in peri-wound CD31+ blood vessel density or numbers. (A) Representative high-power immunohistochemistry images showing CD31+ blood vessels and quantification of (B) CD31+ blood vessel density in the peri-wound areas. (n=5, 2-3 HPF/animal). (C) Representative flow cytometry plots displaying podoplanin on the y axis and CD31+ on the x axis. (D) Quantification of CD31+ BECs as a percentage of CD45- cells (n=3).
